# Comparative Profiling of Yeast Communities in Kefir Grains and Liquid Kefir Using ITS Amplicon Next‐Generation Sequencing

**DOI:** 10.1155/ijfo/2572378

**Published:** 2026-05-08

**Authors:** Kurniawan, Tiana Milanda, Sri Agung Fitri Kusuma

**Affiliations:** ^1^ Doctoral Program, Faculty of Pharmacy, University of Padjadjaran, Bandung, West Java, Indonesia, unpad.ac.id; ^2^ Department of Pharmacist Profession, Faculty of Health Sciences, University of Darussalam Gontor, Ponorogo, East Java, Indonesia; ^3^ Department of Biology Pharmacy, Faculty of Pharmacy, University of Padjadjaran, Bandung, West Java, Indonesia, unpad.ac.id

**Keywords:** biofilm-associated yeasts, fermentation ecology, kefir microbiome, next-generation sequencing, yeast diversity

## Abstract

Kefir is a fermented dairy beverage produced by a complex microbial consortium of bacteria and yeasts coexisting within a polysaccharide–protein matrix known as kefir grains. Although the bacterial communities are well‐characterized, the distribution of yeast taxa, specifically those transitioning into the fermented liquid phase, remains insufficiently described. This study is aimed at characterizing and comparing the fungal community composition in both the kefir grains and the resulting fermented liquid. Using internal transcribed spacer (ITS) amplicon sequencing on samples from a traditionally propagated culture, we identified 122 yeast taxa in the grains and 221 taxa in the liquid fraction, with 182 taxa shared between both habitats. Crucially, taxonomic profiling of the liquid phase revealed a high diversity of yeasts, with *Saccharomyces cerevisiae*, *Kluyveromyces marxianus*, and *Pichia kudriavzevii* being significantly more abundant compared with the grain matrix. In contrast, grain samples were relatively enriched in *Pichia fermentans*. Several taxa maintained moderate abundance across both fractions, suggesting ecological persistence during fermentation. These patterns reflect the spatial distribution of yeast taxa within the system rather than functional specialization. Although based on a single culture without biological replication, these findings provide a detailed descriptive map of the kefir mycobiome, distinguishing dominant fermentation‐associated yeasts in the liquid phase from low‐abundance environmental fungi.

## 1. Introduction

Kefir is a traditional fermented milk beverage characterized by its unique acidic, slightly alcoholic, and effervescent nature. It is produced through the fermentation of milk by kefir grains, a complex, symbiotic consortium of microorganisms embedded within a resilient polysaccharide–protein matrix known as kefiran [1, 2]. The nutritional profile of kefir is superior to raw milk, featuring predigested proteins, bioactive peptides, essential vitamins (such as B1, B12, and K2), and organic acids produced during fermentation. These components contribute to its widely documented health‐promoting properties, including potent antimicrobial activity against pathogens, immunomodulatory effects that enhance the host immune response, and significant modulation of the gut microbiota, which supports gastrointestinal homeostasis [3, 4].

The microbial architecture of kefir is dominated by a stable community of bacteria and yeasts. The bacterial fraction primarily comprises the phyla Firmicutes and Proteobacteria, with a dominance of lactic acid bacteria (LAB) such as *Lactobacillus* (notably *Lactobacillus kefiranofaciens*), *Lactococcus*, and *Leuconostoc*, alongside acetic acid bacteria (AAB) like *Acetobacter* [5, 6]. These bacteria are fundamental to the fermentation process; LAB are responsible for lactose conversion into lactic acid, lowering the pH and contributing to preservation, whereas AAB produce organic acids and contribute to the beverage′s complex aroma profile.

Although the bacterial communities have been extensively profiled, the yeast component representing the “mycobiome” is equally vital but often receives less attention. Yeasts, primarily belonging to the phyla Ascomycota and Basidiomycota, play critical roles in fermentation dynamics. Genera such as *Saccharomyces*, *Kluyveromyces*, and *Pichia* are essential for the production of ethanol and carbon dioxide (CO_2_), which provide kefir′s characteristic “fizz.” Furthermore, yeasts secrete a diverse array of volatile organic compounds (VOCs), including esters and higher alcohols, which define the sensory identity of the beverage [7]. Crucially, yeasts exist in a metabolic syntrophy with LAB; they provide essential growth factors like amino acids and vitamins for the bacteria, whereas LAB acidify the environment, favoring yeast growth. This synergy is also structural; yeasts are integral to the formation and stability of the kefir grain biofilm, acting as anchors within the kefiran matrix [8].

Despite their importance, the spatial distribution of yeast taxa between the grain‐associated biofilm and the surrounding fermented liquid remains poorly understood. Common species such as *Saccharomyces cerevisiae*, *Kluyveromyces marxianus*, and *Pichia* spp. are frequently reported at varying abundance levels, yet it is unclear whether certain taxa are specialized for the grain niche or the planktonic liquid phase. High‐throughput sequencing of fungal internal transcribed spacer (ITS) regions has enabled detailed, culture‐independent profiling of yeast diversity [9]. However, few studies have directly compared paired samples of grains and liquid at species‐level resolution. This gap limits our understanding of niche‐specific adaptation and the potential functional roles of specific yeast taxa.

This study employs ITS amplicon‐based next‐generation sequencing (NGS) to provide a comparative, high‐resolution description of yeast biodiversity in kefir grains and liquid kefir. By framing yeast diversity through a spatially explicit perspective, this work seeks to (i) characterize yeast taxonomic composition and relative abundance patterns, (ii) identify taxa shared between grain and liquid fractions, and (iii) describe habitat‐associated distribution patterns. Although the study is a descriptive assessment of a single fermentation context, it provides a framework for distinguishing dominant fermentation‐associated organisms from transient fungi, thereby improving the ecological interpretation of kefir mycobiome datasets.

## 2. Methods

### 2.1. Sample Collection and Kefir Preparation

The kefir samples were prepared through a controlled fermentation process using a specific substrate and grain ratio. Fresh kefir grains were obtained from Aracaki (Bogor, Indonesia), a traditional kefir producer. The fermentation substrate consisted of a mixture of fresh milk and cheese whey (1 : 1 ratio). The cheese whey was sourced from Mazaraat Artisan Cheese (Yogyakarta, Indonesia), derived from a high‐quality cheese‐making process to ensure a nutrient‐rich medium for microbial growth.

The fermentation was initiated by inoculating the milk‐whey substrate with 5% (*w*/*v*) kefir grains. The process was performed under standardized artisanal conditions at ambient temperature (25°C–28°C) for a duration of 24 h. Following the completion of the fermentation cycle, the kefir grains were separated from the resulting liquid fraction (liquid kefir) using a sterile nylon mesh (2‐mm pore size) under aseptic conditions, following protocols adapted from recent kefir microbiome studies [5, 10]. All samples consisting of both the separated grains and the fermented liquid were collected into sterile polypropylene tubes, immediately frozen at −20°C, and processed within 48 h to ensure the integrity of the microbial community and minimize taxonomic shifts [11].

### 2.2. DNA Extraction

Total genomic DNA was extracted from 250 mg of kefir grain and 1 mL of liquid kefir using the DNeasy PowerFood Microbial Kit (Qiagen, Germany) following the manufacturer′s protocol, with modifications to improve fungal DNA recovery as described by [12]. Briefly, mechanical disruption was performed using 0.5‐mm zirconia beads in a bead‐beater (3 × 60 − s cycles) to ensure efficient lysis of yeast cells embedded in the kefir grain matrix. DNA purity and concentration were measured using a NanoDrop 2000 spectrophotometer (Thermo Fisher Scientific, United States), and integrity was confirmed by 1% agarose gel electrophoresis.

### 2.3. Amplicon Library Preparation and Sequencing

The fungal internal transcribed Spacer 2 (ITS2) region was amplified using primer pair ITS3 (5 ^′^‐GCATCGATGAAGAACGCAGC‐3 ^′^) and ITS4 (5 ^′^‐TCCTCCGCTTATTGATATGC‐3 ^′^), targeting a broad spectrum of yeasts and filamentous fungi . PCR amplification was carried out in triplicate 25‐*μ*L reactions using Phusion High‐Fidelity DNA Polymerase (New England Biolabs, United States). Amplicons were purified with AMPure XP beads (Beckman Coulter, United States) and quantified using a Qubit dsDNA HS Assay Kit (Invitrogen, United States). Indexed libraries were prepared according to Illumina′s 16S/ITS Metagenomic Sequencing Library Preparation protocol and sequenced on an Illumina MiSeq platform (2 × 300 − bp paired − end) at Genetika Sains Indonesia.

### 2.4. Bioinformatics and Taxonomic Assignment

Raw paired‐end reads were processed in QIIME2 v2023.5. Primer sequences were trimmed using Cutadapt [13], and reads were denoised, merged, and chimera‐checked using DADA2 [14]. High‐quality amplicon sequence variants (ASVs) were taxonomically assigned using the UNITE v9.0 database [15] with a 99% similarity threshold.

### 2.5. Community and Statistical Analyses

Relative abundance profiles were generated at phylum, family, genus, and species levels. Dominance levels were categorized as high (≥ 20%), medium (5%–20%), or low (< 5%) relative abundance [16]. Shared and unique yeast species between kefir grains and liquid kefir were visualized using Venn diagrams generated in the matplotlib-venn Python package. Alpha diversity (Shannon, Simpson) and beta diversity (Bray–Curtis dissimilarity) metrics were calculated in QIIME2. Statistical differences in community composition were tested by PERMANOVA with 999 permutations. All statistical analyses were conducted in R v4.3.2 using vegan and ggplot2 packages.

## 3. Results and Discussion

Because the present study was based on paired kefir grain and liquid samples derived from a single fermentation batch without biological replication, the observations presented below should be interpreted as descriptive patterns of fungal community composition rather than definitive ecological mechanisms. The results therefore are aimed at providing an exploratory overview of yeast biodiversity within the kefir fermentation system and at identifying potential distribution patterns between the structured grain matrix and the surrounding liquid environment.

**Figure 1:**
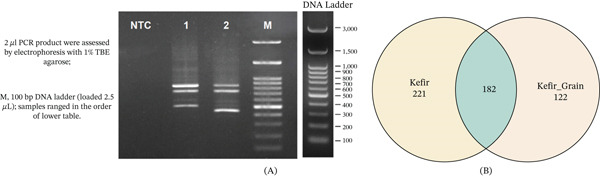
Characterization and distribution of the kefir mycobiome. (A) Agarose gel electrophoresis (1% TBE) of ITS nested PCR amplicon libraries from fermented liquid (Lane 1) and grain (Lane 2) samples. Lane M represents the 100‐bp DNA ladder. (B) Venn diagram illustrating the distribution and overlap of yeast operational taxonomic units (OTUs) between the liquid and grain fractions.

Profiling of the fungal community began with nested PCR of the fungal ITS region. Gel electrophoresis of the resulting amplicon libraries from both the liquid kefir and grain fractions showed distinct, well‐defined bands (Figure 1A), confirming successful DNA extraction and targeted amplification of the mycobiome. The sample distribution of yeast taxa, determined via ITS amplicon sequencing, is illustrated in Figure 1B. Taxonomic profiling revealed a high degree of overlap between the two distinct fermentation environments. The visualization of the microbial communities identified via ITS amplicon sequencing is presented in the combined and Figure 1, illustrating both the initial molecular characterization and the broad taxonomic distribution.

A critical finding of this study is the high number of shared yeast taxa (*n* = 182) detected in both the grain and liquid fractions. This substantial overlap highlights the intense microbial exchange that defines the fermentation process. Kefir grains function as a biological anchor, a complex biofilm that constantly sheds organisms into the dynamic liquid medium. These shared taxa likely represent the core, “persistent mycobiome” [5, 7], composed of yeasts that are adapted to coexist syntrophically with the bacteria in both the sessile biofilm‐like state and the planktonic liquid phase. The presence of these species across both habitats underscores that, in a traditional batch‐type system, the liquid fraction essentially maps the dominant biodiversity from the surface of the grains. Although the liquid phase can host an additional 39 distinct low‐abundance taxa (Figure 1B), these likely represent transient environmental fungi that find a brief niche in the dynamic, nutrient‐rich substrate, rather than key players in the core fermentation profile.

**Figure 2:**
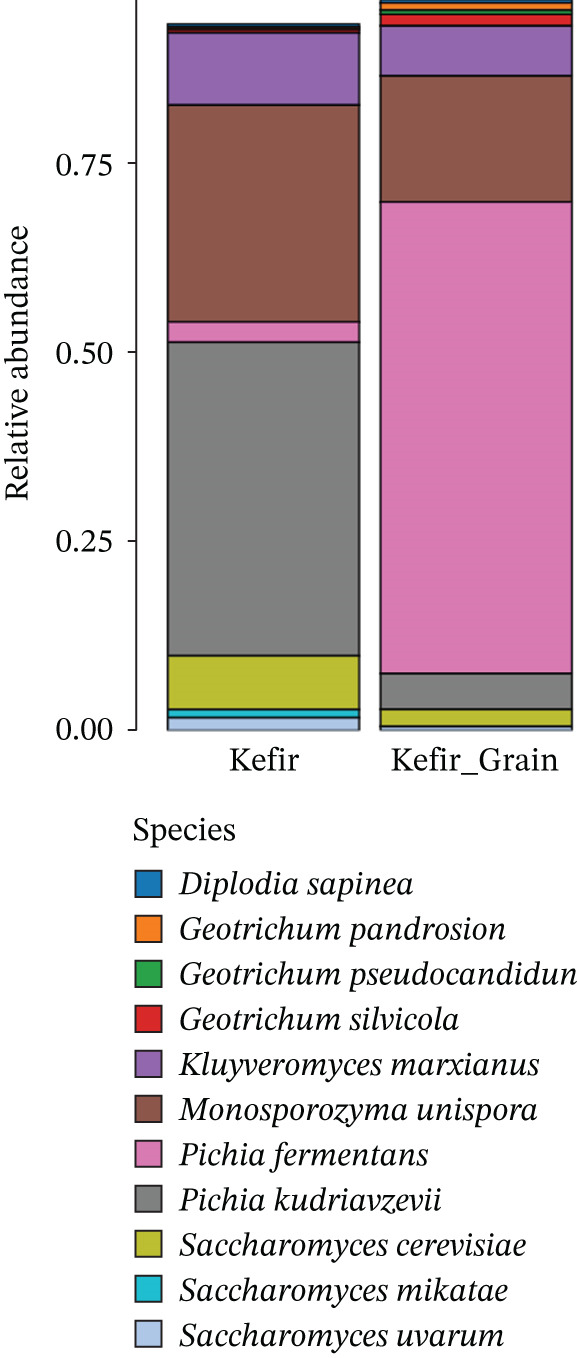
Relative abundance of yeast species in fermented liquid kefir and kefir grains. Stacked bar plots illustrating the taxonomic distribution at the species level for both fermentation fractions.

The NGS analysis revealed a diverse yeast community in both kefir grains and liquid kefir, with marked differences in species composition and relative abundance between the two matrices. The Top 10 most abundant yeast species exhibited distinct distribution patterns, indicating that the structural and physicochemical properties of the fermentation matrix play a pivotal role in shaping yeast biodiversity. Analysis of the most abundant yeast taxa showed clear differences in relative abundance patterns between kefir grains and liquid kefir (Figure 2).

In kefir grains, taxa such as *S*. *cerevisiae*, *K*. *marxianus*, and *Kazachstania unispora* were among the dominant species. In contrast, the liquid fraction exhibited higher relative abundance of taxa including *Candida kefyr* and *Pichia fermentans*. These patterns may reflect differences in environmental conditions between the structured grain matrix and the surrounding liquid phase, although further replicated studies would be required to confirm potential ecological specialization. These taxa are recognized for their high fermentative capacity, metabolic versatility, and potential contributions to kefir′s flavor, texture, and probiotic functionality [6, 17]. The consistent prevalence of *K. marxianus* and *K*. *unispora* in the grain fraction suggests a stable, long‐term symbiotic association with LAB embedded within the kefir grain biofilm matrix. Conversely, the liquid kefir fraction displayed a different dominance profile, with *S. cerevisiae* remaining prevalent but accompanied by higher relative abundances of *C*. *kefyr* and *P*. *fermentans*. The enrichment of *Candida* and *Pichia* species in the liquid phase is consistent with their ability to thrive in unstructured, nutrient‐rich environments, characteristic of the suspended fraction during fermentation [18, 19]. Such shifts suggest that the liquid environment favors opportunistic, fast growing yeasts adapted to planktonic growth.

The spatial organization of these yeast communities is intrinsically linked to the physical architecture of the fermentation system. Kefir grains act as a stable reservoir for structured yeast communities due to their complex, three‐dimensional biofilm‐like composition. Structurally, the grains are composed of a resilient matrix of kefiran, a branched water‐soluble glucogalactan interspersed with proteins and lipids [2]. This matrix provides a heterogenous environment with distinct microniches; the outer layers are typically more aerobic, favoring oxidative yeasts, whereas the dense inner core supports anaerobic fermentation. This structural stability allows the grains to maintain a consistent “microbial memory” across generations of propagation, even as they shed organisms into the liquid phase. Within this complex ecosystem, the high‐sensitivity sequencing employed in this study also identified a diverse group of low‐abundance but consistently present species, such as *Diplodia sapinea* and *Monosporozyma unispora*. Although these taxa do not dominate the fermentation in terms of biomass, their persistent detection across both fractions suggests they are not merely transient contaminants. Instead, their presence may indicate a broader ecological tolerance or supportive roles within the consortium. For instance, such minor taxa can contribute to the “functional redundancy” of the system, potentially offering enzymatic capabilities that assist in breaking down complex milk components or producing secondary metabolites that stabilize the symbiotic community against environmental stress [7, 8].

The dominance patterns of the identified yeast species, showing the distribution differences between the two matrices, are summarized in Table 1.

**Table 1:** Relative abundance (%) of dominant yeast species in kefir grains and liquid kefir.

Species	Kefir grains (%)	Liquid kefir (%)	Dominance description
*Pichia fermentans*	~65.0	~5.0	Predominant in kefir grains; associated with biofilm‐like matrix
*Pichia kudriavzevii*	~3.0	~60.0	Predominant in liquid kefir; fermentative planktonic yeast
*Kluyveromyces marxianus*	~28.0	~15.0	Abundant in both fractions; relatively higher in grains
*Saccharomyces cerevisiae*	~5.0	~10.0	Moderate abundance; relatively enriched in liquid kefir
*Diplodia sapinea*	~4.0	~4.0	Comparable abundance across both habitats
*Monosporozyma unispora*	~3.0	~3.0	Intermediate abundance; broad ecological tolerance
*Geotrichum pandrosion*	~6.0	~1.0	Enriched in kefir grains; potentially surface‐associated
*Geotrichum silvicola*	~2.0	~1.0	Minor taxon; more frequently detected in grains
*Geotrichum pseudocandidum*	~1.0	< 1.0	Minor taxon in both fractions
*Saccharomyces uvarum*	< 1.0	< 1.0	Low‐abundance species
*Saccharomyces mikatae*	< 1.0	< 1.0	Low‐abundance species

As illustrated in the data above, *P*. *fermentans* showed high relative abundance in kefir grains but occurred at much lower levels in the liquid fraction, whereas *Pichia kudriavzevii* displayed the opposite pattern. Such contrasting abundance profiles may suggest potential habitat‐associated preferences among yeast taxa. However, given the exploratory nature of the present study, these observations should be interpreted as indicative distribution patterns rather than definitive evidence of ecological specialization.

Yeast communities in kefir grains and liquid kefir displayed distinct dominance patterns, indicating habitat‐associated ecological partitioning. *P*. *fermentans* dominated kefir grains (~65%) but was scarce in the liquid fraction, whereas *P*. *kudriavzevii* was highly enriched in liquid kefir (~60%) and occurred at low abundance in grains. This pattern suggests niche specialization driven by differences in physical structure and nutrient availability between the grain matrix and liquid environment [5, 18]. Comparable spatial differentiation has been reported in kefir from Europe and the Caucasus, where biofilm‐associated yeasts preferentially colonize grains and fermentative yeasts dominate the liquid phase; however, most previous studies relied on culture‐dependent methods or genus‐level resolution [4, 17]. By applying ITS‐based NGS, the present study provides species‐level evidence supporting habitat‐driven yeast differentiation. *K*. *marxianus* and *S*. *cerevisiae* were detected in both habitats, consistent with their metabolic versatility and frequent occurrence across diverse fermented foods [20]. In contrast, taxa such as *D*. *sapinea*, *M*. *unispora*, and *Geotrichum* spp. showed lower to moderate abundance but stable presence, suggesting broader ecological tolerance or supportive roles within the kefir ecosystem [21] Overall, this paired‐sample approach addresses a gap in international kefir microbiome research by resolving yeast community structure separately in grains and liquid kefir. Nevertheless, the findings remain descriptive and context‐specific, highlighting the need for multiorigin sampling and functional analyses to generalize these ecological patterns [6].

Venn diagram analysis further supported these observations, revealing a stable core microbiota shared between grains and liquid kefir, alongside unique taxa restricted to each fraction. The shared taxa likely represent the essential functional core of the kefir microbiome, whereas fraction‐specific taxa reflect ecological niche specialization and microbial succession across fermentation stages. Overall, these findings indicate that kefir grains act as a stable reservoir for structured yeast communities, whereas the liquid kefir fraction promotes dynamic microbial interactions and compositional turnover, both contributing to kefir′s microbial diversity and functional properties.

**Figure 3:**
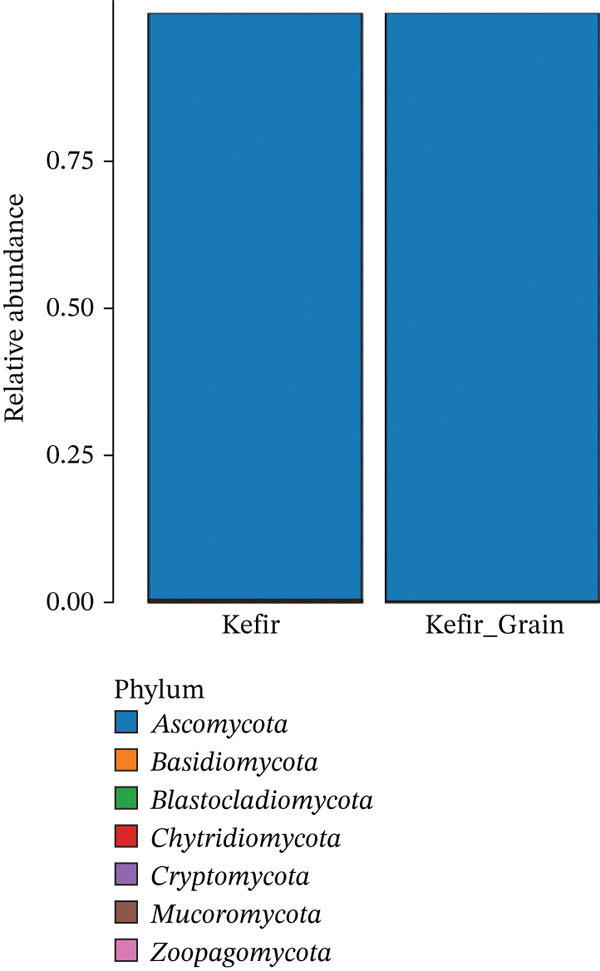
Relative abundance of fungal phyla in fermented liquid kefir and kefir grains. Stacked bar chart showing the taxonomic distribution of fungal communities at the phylum level for both samples.

To provide a broader taxonomic context of the kefir mycobiome, the community composition was further evaluated at the higher taxonomic rank, as illustrated in Figure 3.

As shown in Figure 3, the relative abundance analysis revealed that both kefir grains and liquid kefir were overwhelmingly dominated by members of the phylum Ascomycota, representing nearly 100% of the detected fungal community. This consistent dominance across both sample types indicates a highly specialized yeast community structure, aligning with previous reports that Ascomycota, particularly within the order Saccharomycetales, are the principal fungal constituents in fermented dairy ecosystems [22]. Other fungal phyla such as Basidiomycota, Blastocladiomycota, Chytridiomycota, Cryptomycota, Mucoromycota, and Zoopagomycota were either absent or present at negligible levels, suggesting that these taxa are unable to withstand the selective pressures inherent in the kefir fermentation environment or occur only as transient contaminants. The acidic pH, lactose‐rich substrates, and predominantly microaerophilic to anaerobic niches characteristic of kefir favor the proliferation of *Ascomycetous* yeasts, including *Saccharomyces*, *Kluyveromyces*, and *Pichia* [5]. This pronounced dominance underscores the ecological filtering imposed by the kefir fermentation matrix, which promotes metabolically versatile Ascomycota capable of ethanol production, organic acid metabolism, and biofilm formation [1, 2]. The lack of significant Basidiomycota representation contrasts with other fermented foods, such as kombucha or cocoa fermentation, where this phylum can contribute substantially to the fungal community [23], further highlighting kefir′s distinct fungal ecology.

**Figure 4:**
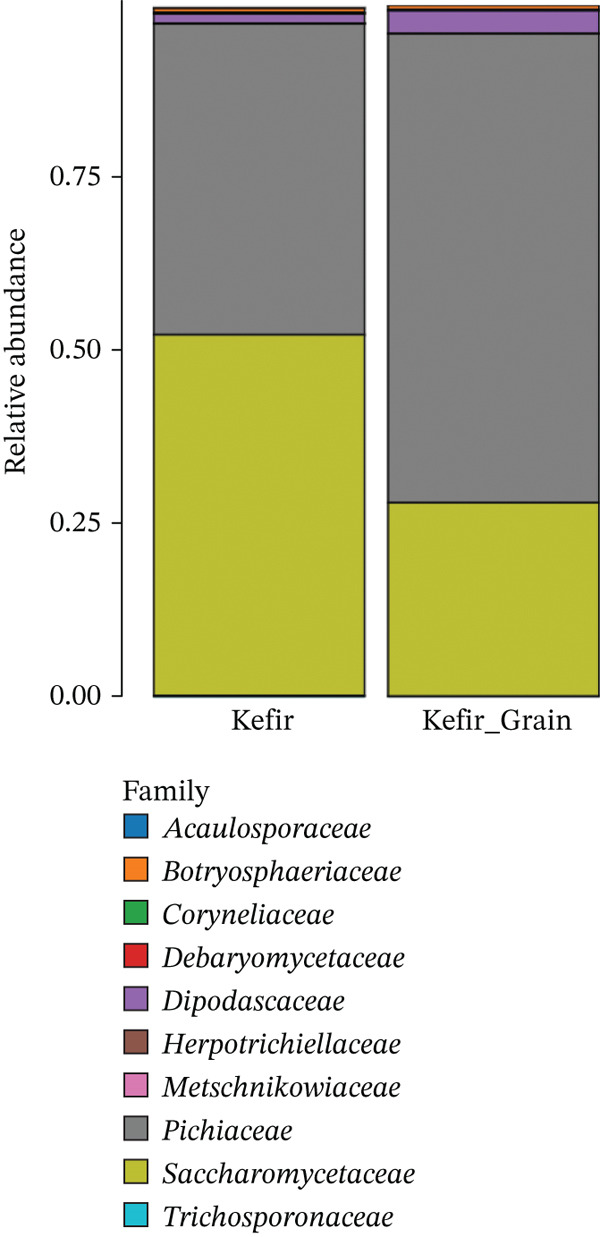
Relative abundance of fungal families in fermented liquid kefir and kefir grains. Stacked bar chart illustrating the distribution of fungal families detected in both fractions.

At a finer taxonomic resolution, the distribution of fungal families was analyzed to identify the core groups driving the fermentation, with the comparative results presented in Figure 4.

As shown in Figure 4, the fungal communities at the family level in both kefir grains and liquid kefir were overwhelmingly dominated by Saccharomycetaceae and Pichiaceae, which together accounted for the majority of relative abundance. Saccharomycetaceae was markedly more prevalent in the liquid kefir fraction, whereas Pichiaceae exhibited higher representation in the grain fraction. This distribution is consistent with the ecological traits of their representative genera. *Saccharomyces* species are typically fast growing, planktonic fermenters well adapted to nutrient‐rich liquid environments, whereas *Pichia* species are often biofilm‐associated and more resilient within structured matrices such as kefir grains [2, 5, 24]. Other families detected at lower abundances included Metschnikowiaceae, Trichosporonaceae, and Dipodascaceae, which may nonetheless contribute to niche‐specific metabolic interactions. For instance, *Metschnikowia* species are known to produce extracellular enzymes and antimicrobial compounds, potentially influencing microbial succession during fermentation [25]. Additionally, Botryosphaeriaceae and Coryneliaceae families, more typically associated with plant or environmental sources, likely represent transient environmental contaminants or minor constituents with limited functional roles in kefir ecosystems. Overall, the distribution pattern suggests that kefir grains serve as reservoirs for *Pichia-dominated* consortia, potentially supporting biofilm resilience and structural integrity, whereas liquid kefir is enriched in *Saccharomyces-driven* fermentative metabolism. This family‐level partitioning reinforces the concept of spatial niche differentiation between grain and liquid habitats, a phenomenon also observed in other complex fermentations such as sourdough and kombucha [26].

**Figure 5:**
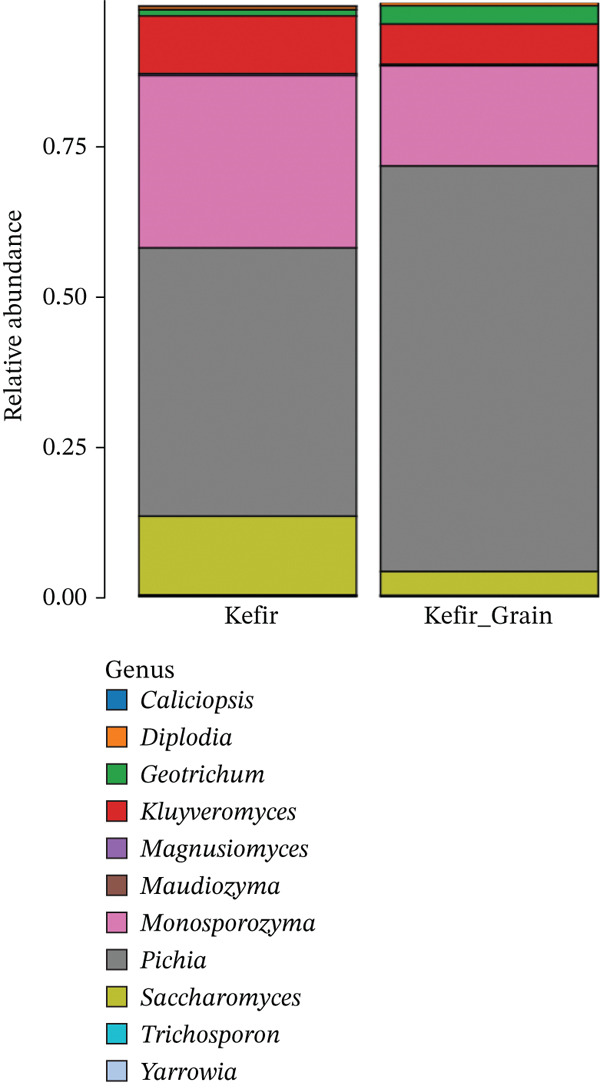
Relative abundance of fungal genera in fermented liquid kefir and kefir grains. Stacked bar chart showing the taxonomic distribution of fungal communities at the genus level.

The microbial community was further resolved to the genus level to pinpoint the specific taxa contributing to the fermentation profile, as detailed in Figure 5.

As shown in Figure 5, the genus‐level fungal composition of kefir grains and liquid kefir reveals distinct patterns of niche differentiation. *Pichia* dominated the grain fraction, whereas *Saccharomyces* was the primary genus in the liquid fraction. This shift in dominance aligns with previous findings that *Pichia* species are well adapted to structured, biofilm‐like matrices due to their capacity for cell‐surface adhesion and biofilm formation [21, 27], whereas *Saccharomyces* thrives in nutrient‐rich, planktonic environments, optimizing ethanol fermentation and rapid population growth [20, 30]. *Monosporozyma* was detected at high relative abundance across both fractions, indicating notable ecological versatility and potential involvement in both sessile and planktonic phases of kefir fermentation. Other genera, such as *Kluyveromyces* and *Geotrichum*, although present at lower levels, hold functional importance. For instance, *K*. *marxianus* is recognized for its *β*‐galactosidase activity and thermotolerance, facilitating lactose hydrolysis in dairy fermentations [28, 31]. Genera including *Diplodia*, *Magnusiomyces*, and *Trichosporon* occurred at low abundances, suggesting they may function as transient or minor constituents without central fermentative roles, yet potentially contributing secondary metabolites or influencing microbial interactions. The coexistence of dominant and rare taxa across both environments supports the concept of a layered functional network in kefir ecosystems, where primary fermentation is driven by abundant genera, whereas low‐abundance members enhance flavor complexity, stability, and community resilience [2, 5, 29, 32].

Based on Figures S1–S3, the heatmap analyses consistently demonstrate clear habitat‐associated structuring of yeast communities across species, family, and genus levels between kefir grains and liquid kefir. At the species level (Figure S1), strong clustering highlights the dominance of *P*. *fermentans* in kefir grains and *P*. *kudriavzevii*, *S*. *cerevisiae*, and *K*. *marxianus* in the liquid fraction, indicating fine‐scale ecological partitioning that is often unresolved in culture‐based studies [5, 18, 33]. The family‐level heatmap (Figure S2) further supports this pattern, with Pichiaceae prevailing in the grain‐associated biofilm matrix, whereas Saccharomycetaceae are enriched in the liquid phase, reflecting differential adaptation to structured versus planktonic environments, as previously suggested in European kefir surveys [4, 17, 34]. At the genus level (Figure S3), the co‐occurrence of *Pichia*, *Saccharomyces*, and *Kluyveromyces* across both habitats underscores the presence of a shared core microbiota, whereas differences in relative intensity reveal niche specialization rather than simple presence–absence patterns. Compared with international kefir studies, which commonly report yeast diversity at the genus level or aggregate grains and liquid samples into a single microbiome profile [5, 31, 35, 36], this study provides a more spatially resolved view of yeast biodiversity using ITS‐based NGS. The clear separation observed across all taxonomic ranks addresses a critical gap in global kefir microbiome research, where habitat‐specific yeast ecology remains underexplored. Nonetheless, similar to most international reports, the present analysis remains descriptive, emphasizing the need for future multiomics and multiorigin studies to link these taxonomic patterns with functional and metabolic roles across diverse kefir production systems. Heatmap analysis provided a detailed visualization of fungal community distribution across species, family, and genus levels in kefir grains and liquid kefir (Figures S1–S3).

The Sankey diagrams (Figures S4–S5) provide an integrated visualization of taxonomic flow from kingdom to species level, highlighting structural differences between kefir grain–associated and liquid kefir fungal communities while confirming dominance patterns observed in previous analyses. In both fractions, the fungal community was overwhelmingly dominated by Ascomycota, with Saccharomycetaceae and Pichiaceae representing the principal families structuring the kefir mycobiome. The grain‐associated community was primarily driven by *P*. *fermentans*, reflecting adaptation to the structured polysaccharide‐rich grain matrix, whereas the liquid fraction exhibited a more distributed composition characterized by fermentative yeasts such as *S*. *cerevisiae*, *K*. *marxianus*, and *K*. *unispora*. Several taxa, including *M*. *unispora* and *D*. *sapinea*, displayed moderate representation across both habitats, suggesting ecological flexibility between biofilm‐associated and planktonic environments. Low‐abundance genera such as *Geotrichum*, *Magnusiomyces*, and *Yarrowia* likely represent peripheral community members contributing secondary metabolic functions rather than primary fermentation roles. Alpha diversity analysis further indicated higher richness and evenness in liquid kefir compared with kefir grains across multiple indices (observed species, Chao1, ACE, Shannon, Simpson, Inverse Simpson, and Fisher). Although not visualized as a standalone figure, these metrics consistently showed that the liquid phase supports a more heterogeneous yeast assemblage, whereas the structurally constrained grain matrix favors dominance by fewer matrix‐adapted taxa. This pattern supports the interpretation that kefir grains function as a selective microbial reservoir, whereas the liquid environment promotes coexistence of multiple fermentative yeasts and increased ecological variability during fermentation [2, 5, 38, 38]. The taxonomic distribution of dominant yeast taxa across fermentation matrices is illustrated using Sankey diagrams (Figures S4–S5).

## 4. Conclusions

This study demonstrates a clear spatial differentiation of yeast communities between kefir grains and liquid kefir based on ITS amplicon sequencing. Yeasts associated with structured, biofilm‐like environments, particularly *P*. *fermentans*, were more prevalent in kefir grains, whereas the liquid fraction was enriched in fermentative, planktonic yeasts such as *S*. *cerevisiae* and *K*. *marxianus*. A subset of taxa was shared between both matrices, indicating partial overlap and ecological continuity within the kefir fermentation system.

These findings provide a taxonomic framework describing the organization of yeast communities in distinct kefir microenvironments. However, as the analysis was limited to a single fermentation batch and relied on relative abundance inferred from ITS‐based sequencing, functional activity, temporal dynamics, and strain‐level variation could not be assessed. Consequently, the results should be interpreted as descriptive of community structure rather than direct indicators of metabolic function or health effects. Future studies incorporating multiple batches, time‐series sampling, and functional approaches such as metabolomics or metatranscriptomics will be required to further elucidate the ecological and functional roles of yeasts in kefir fermentation.

## Funding

This study was supported by the Universitas Padjadjaran (Contract N0. 4303/B3/DT.03.08/2025 and 3927/UN6.RKT/HK.07.00) and Universitas Darussalam Gontor.

## Conflicts of Interest

The authors declare no conflicts of interest.

## Supporting information


**Supporting Information** Additional supporting information can be found online in the Supporting Information section. Figures S1–S5 show detailed fungal community composition analyses, including heatmap and Sankey diagrams.

## Data Availability

The sequencing datasets generated during this study are publicly available in the Zenodo repository: https://zenodo.org/records/19046637.
