# Supplementary material for: Comparative Profiling of Yeast Communities in Kefir Grains and Liquid Kefir Using ITS Amplicon Next‐Generation Sequencing
Source: Int J Food Sci. 2026 May 8;2026:2572378. doi: 10.1155/ijfo/2572378 (PMC13154769; doi:10.1155/ijfo/2572378)
Supplement: Supplementary file 1 — Supporting Information Additional supporting information can be found online in the Supporting Information section. Figures S1–S5 show detailed fungal community composition analyses, including heatmap and Sankey diagrams. [file IJFO-2026-2572378-s001.docx]

Supplementary Material

# Supplementary Figures and Tables

## Supplementary Figures

**Suplementary Figure S1-S3**. Heatmap of fungal distribution at species, family, and genus **levels.** The heatmaps illustrate the log10-transformed relative abundance and hierarchical clustering of fungal communities detected in fermented liquid kefir and kefir grains.


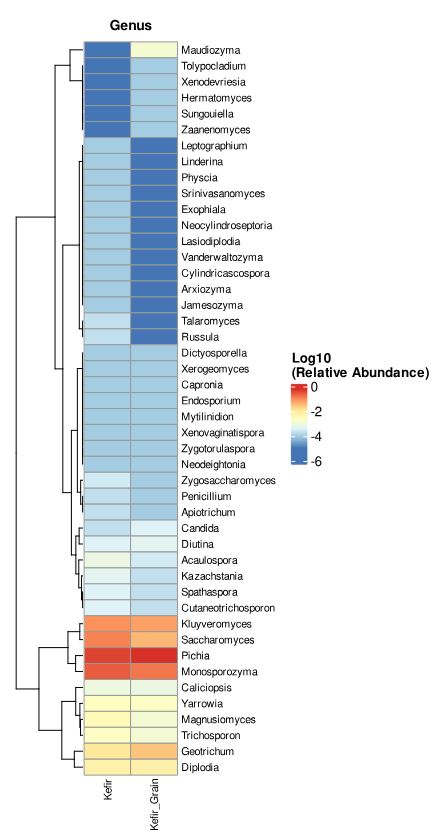

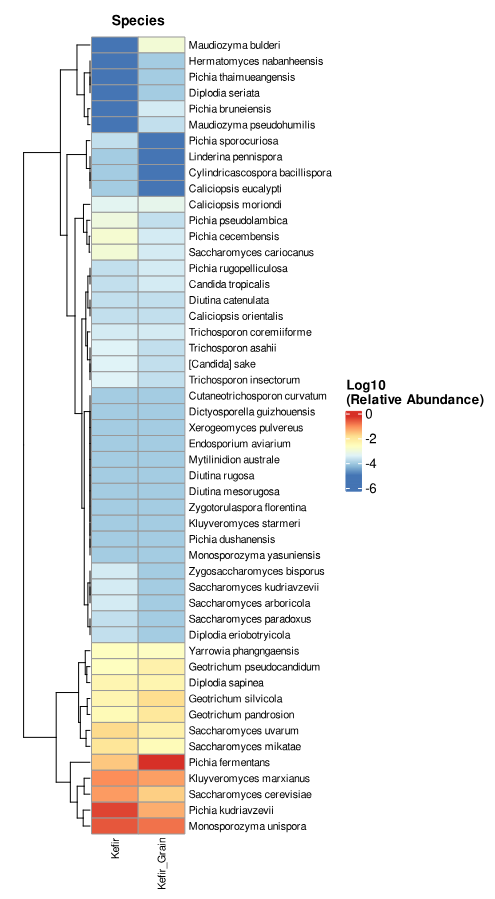

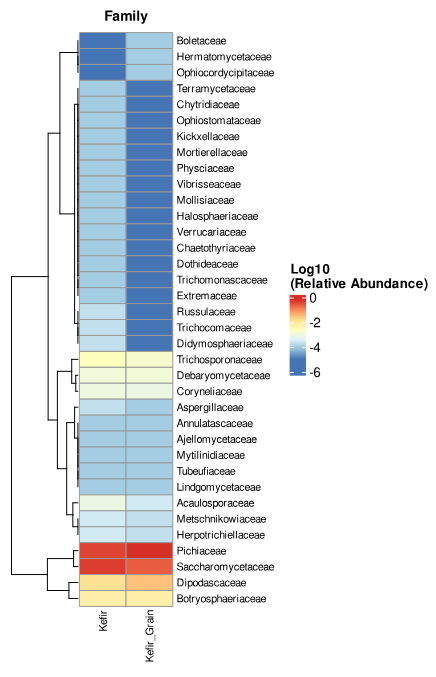


(S1) (S2) (S3)

Supplementary Figure S1. Heatmap of fungal species relative abundance in fermented liquid kefir and kefir grains.

Supplementary Figure S2. Heatmap of fungal family relative abundance in fermented liquid kefir and kefir grains.

Supplementary Figure S3. Heatmap of fungal genus relative abundance in fermented liquid kefir and kefir grains.

**Suplementary Figure S4, S5**. Sankey diagrams of fungal taxonomic distribution in fermented liquid kefir and kefir grains. The diagrams illustrate the hierarchical composition of fungal communities from domain to species level, with flow width representing relative abundance.


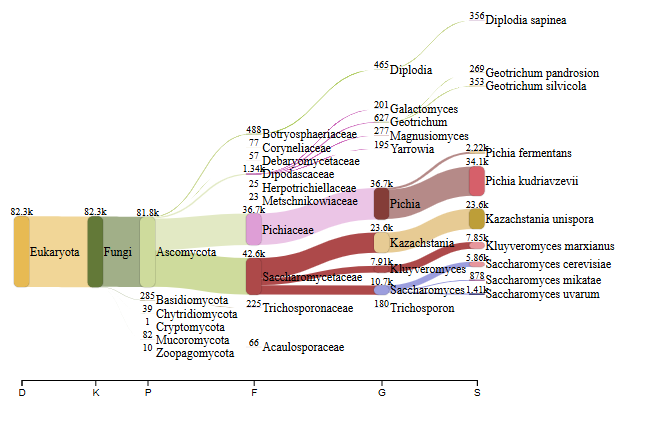

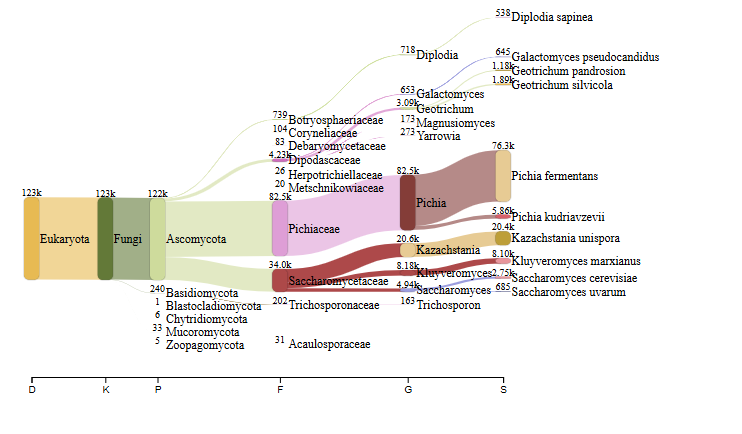


**(S4) (S5)**

Supplementary Figure S4. Sankey diagram illustrating fungal taxonomic distribution in kefir grains.
Supplementary Figure S5. Sankey diagram illustrating fungal taxonomic distribution in liquid kefir.
